# Books and Pamphlets Received

**Published:** 1888-01

**Authors:** 


### BOOKS AND PAMPHLETS RECEIVED.

*The Principles of Antiseptic Methods Applied to Practice;* by Dr. Paul, Bar Accoucheur to, formerly Interne in the Maternity Hospital, Paris, etc. Philadelphia · P. Blakiston, Son & Co.

The work contains 174 large octavo pages and treats the subject ably under the following heads or chapters:

1. Relation of the Germ Theory to the Puerperium.
2. Antiseptic Method and Agents.
3. Influence of Antiseptics on Puerperal Epidemics.
4. Disinfection as an Antiseptic Measure.
5. Hygeine of the Peurperium.
6. Antisepsis During Labor.
7. Antisepsis During the Third Stage of Labor.
8. Antisepsis During the Puerperium.
9. Antisepis in Catheterization.
10. Antiseptic Method in Rupture of the Uterus.
11. Antisepsis in Cæsarian Operation.

Lastly, Antisepsis to the Umbilicus and in Ophthalmia Neonatorium.

*Differential Diagnosis*—A Manual of the Comparative Semeiology of the More Important Diseases, by F. De Havelland Hall, M.D., Assistant Physician to the Westminster Hospital, London. Third American Edition thoroughly revised and greatly enlarged, edited by Frank Woodburry, M.D., Professor of Therapeutics and Materia Medica, and of Clinical Medicine in the Medico-Chirurgical College, etc., Philadelphia. D. G. Brinton. 1887.

A work of 250 octavo pages well written and ably gotten up. The subjects are treated of under the principal heads of General and Local Diseases. The former under two chapters: 1. Fevers, including all the prominent and usual types. 2. Diseases of the Blood.

The Local Diseases are considered under five chapters as follows: 1. Diseases of the Nervous System. 2. Diseases of the Respiratory Apparatus. 3. Diseases of the Circulatory Apparatus. 4. Diseases of the Digestive System. 5. Diseases of the Urinary Organs.

*A Treatise on the Physiology of the Earth;* or, the Origin, Growth and Decay of the Earth, with a Monograph on the Tribal Relations of Man. By S. P. Crawford, A. M., M. D., Stockton, Cal. 1887.

A work of 73 pages in which the author gives some peculiar views, treated with marked ability and originality under the following interesting heads: Genesis of the Earth; Solid Matter of the Earth; Animals, Including Man; Man's Mission and Destiny; Tribal Relations of Man; Black Races.

*Treatment of Hemorrhoids* by Injections of Carbolic Acid and Other Substances. By Silas T. Yount, M. D., Physician of St. Elizabeth's Hospital; Member of American Medical Association; Member Indiana State Medical Association; Member of the Tippicanoe County Medical Society. The Echo Music Co., Printers. LaFayette, Indiana. 1887.

This is a very instructive and useful little work on the subject treated.

*Functional Nervous Diseases;* their Causes and their Treatment. Memoirs for the Concourse of 1881–1883. Academie Royal De Medecine De Belgique, with a Supplement on the Anomalies of Refraction of the Eye and the Ocular Muscles; by Geo. T. Stevens, M. D., Ph. D., member of the American Medical Association, of the American Ophthalmological Society; formerly Professor of Ophthalmology and Physiology in the Albany Medical College. D. Appleton & Co., New York.

A volume of 217 large octavo pages, neatly printed, and gotten up with marked ability. It treats learnedly of all nervous diseases, their cause and treatment. We were particularly struck with the discussion upon *Chorea*, Epilepsy, Mental Disorders and Heredity. The work is eminently suggestive and practical upon numerous points, and must prove interesting and very useful to the student and practitioner.

*Lessons in Gynecology.* By William Godell, A.M., M.D., Professor of Clinical Gynecology in the University of Pennsylvania, etc. Third edition thoroughly revised and greatly enlarged, with 112 illustrations. Philadelphia. D. G. Brinton. 1887.

The above work is a valuable addition to the literature of the department of which it treats. It is largely made up of the clinical and didactic lectures delivered to the third year students of the Medical Department of the University of Pennsylvania.

Evidences of the value of this work is found in the rapid sale of the two editions first issued, and now that we have a third edition, revised and improved, there can be no doubt that it will be welcomed and largely patronized by members of the profession everywhere, especially by those who, as teachers or students, are giving attention to gynecological studies.

SANDERS & SONS' EUCALYPTI EXTRACT (*Eucalyptol*).—Apply to Dr. Sanders, Dillon, Iowa, for gratis supplied reports on cures effected at the clincs of the Universities of Bonn and Griefswald.

**Goitre.**—The fluid extract of bannana root is said to cure the worst forms of goitre.

